# 50 years of scanning electron microscopy of bone—a comprehensive overview of the important discoveries made and insights gained into bone material properties in health, disease, and taphonomy

**DOI:** 10.1038/s41413-019-0053-z

**Published:** 2019-05-22

**Authors:** Furqan A. Shah, Krisztina Ruscsák, Anders Palmquist

**Affiliations:** Department of Biomaterials, Sahlgrenska Academy, University of Gothenburg, Gothenburg, Sweden

**Keywords:** Bone, Bone quality and biomechanics

## Abstract

Bone is an architecturally complex system that constantly undergoes structural and functional optimisation through renewal and repair. The scanning electron microscope (SEM) is among the most frequently used instruments for examining bone. It offers the key advantage of very high spatial resolution coupled with a large depth of field and wide field of view. Interactions between incident electrons and atoms on the sample surface generate backscattered electrons, secondary electrons, and various other signals including X-rays that relay compositional and topographical information. Through selective removal or preservation of specific tissue components (organic, inorganic, cellular, vascular), their individual contribution(s) to the overall functional competence can be elucidated. With few restrictions on sample geometry and a variety of applicable sample-processing routes, a given sample may be conveniently adapted for multiple analytical methods. While a conventional SEM operates at high vacuum conditions that demand clean, dry, and electrically conductive samples, non-conductive materials (e.g., bone) can be imaged without significant modification from the natural state using an environmental scanning electron microscope. This review highlights important insights gained into bone microstructure and pathophysiology, bone response to implanted biomaterials, elemental analysis, SEM in paleoarchaeology, 3D imaging using focused ion beam techniques, correlative microscopy and in situ experiments. The capacity to image seamlessly across multiple length scales within the meso-micro-nano-continuum, the SEM lends itself to many unique and diverse applications, which attest to the versatility and user-friendly nature of this instrument for studying bone. Significant technological developments are anticipated for analysing bone using the SEM.

## Introduction

Half a century on since the pioneering work of Boyde and co-workers,^1,2^ the scanning electron microscope (SEM) is now an analytical staple in the assessment of bone microarchitecture in health and disease^3^ and in vivo performance of biomedical implant materials.^4,5^ Beyond the realm of medicine, the SEM is also used regularly in paleoarchaeology and forensic anthropology. The purpose of this review is to highlight key insights gained using the SEM, into bone microstructure and pathophysiology, bone response to various classes of implanted biomaterials, elemental analysis using energy-dispersive X-ray spectroscopy (EDX), examples of SEM in paleoarchaeology, focused ion beam (FIB) techniques for 3D imaging, correlative microscopy and in situ experiments.

**Fig. 1:**
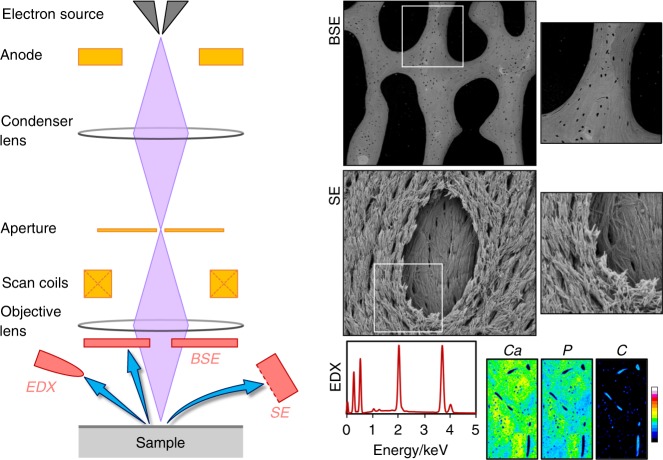
Parts of a scanning electron microscope (SEM) and the typical signals that are recorded from bone. BSE backscattered electrons, SE secondary electrons, EDX energy-dispersive X-ray spectroscopy

Interactions between incident electrons and atoms on the sample surface (and a limited sub-surface volume) generate various signals. These include backscattered electrons (BSEs) and secondary electrons (SEs) that relay compositional and topographical information.^6^ In brief, a finely focused incident electron beam moves across the sample's surface and electrons emitted from each position within the scanned area are collected by a detector (Fig. 1). A conventional SEM is operated at high vacuum conditions that require samples to be clean, dry, and electrically conductive. Most biological systems/materials are non-conductive, and in order to avoid static charge build-up they must be rendered electrically conductive for which various strategies are available, e.g., impregnation with heavy metals, application of thin conductive coatings (Ag, Pt, Pd, C), and use of room-temperature ionic liquids.

SEs are low-energy electrons ejected from the inner shells of the atoms in the sample, as a result of inelastic scattering interactions with the incident electrons. SEs typically originate from within a few nanometres from the sample surface. BSEs are high-energy electrons of the incident beam that are deflected back by very high angles due to elastic scattering interactions with atomic nuclei. BSE *Z*- (atomic number) contrast enables distinguishing between regions on the sample surface having different average atomic numbers. Heavier elements (high *Z*) backscatter electrons more efficiently than lighter elements (low *Z*) and thus appear brighter in the image.

Non-conductive samples can be imaged without modification from their natural state, thus preserving their original characteristics, using an environmental scanning electron microscope (ESEM). The sample chamber is isolated from the electron column using multiple pressure limiting apertures. An imaging gas, e.g., water vapour, is introduced into the sample chamber. Since these gas molecules can scatter the electrons and degrade the electron beam, high vacuum maintained throughout the electron column, while the sample chamber may sustain high-pressure states. Interactions between the primary electron beam and the sample surface release SEs (as in a conventional SEM). These SEs encounter water vapour molecules, generating a cascade of SEs, thus amplifying the original SE signal, which is collected at an electrically biased gaseous secondary electron detector (GSED). BSEs also pass through the gaseous volume and induce additional ionisation and generate amplification. The electrical bias on the GSED drives the positively charged water vapour molecules towards the sample surface, effectively neutralising static charge build-up.

## Bone imaging

**Fig. 2:**
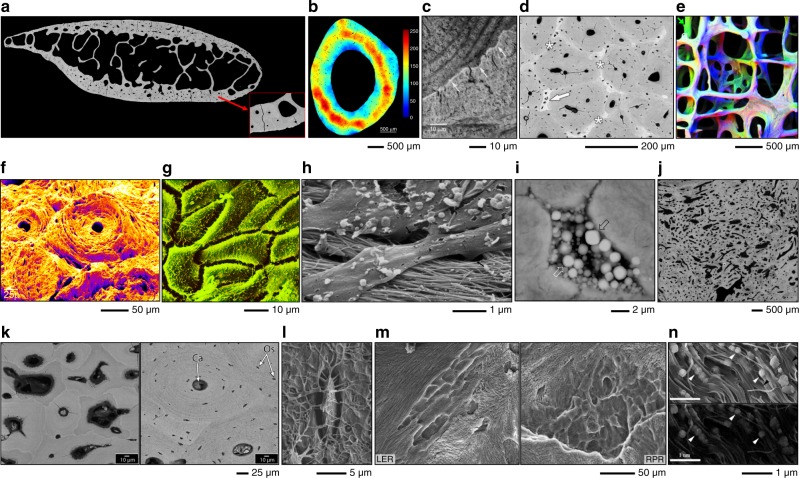
Imaging bone in the SEM. **a** BSE-SEM photomontage of a human rib viewed in cross-section. Local variations in mineralisation density, Haversian canals, resorption spaces, and osteocyte lacunae can be detected. From Bereshiem et al. Adapted with permission from John Wiley and Sons. Copyright 2019 ^7^. **b** Osteocyte lacunar density determined using BSE imaging. From Bach-Gansmo et al.^12^. Adapted with permission from Elsevier. Copyright 2015^12^. **c** A cement line between osteonal bone and interstitial bone. From Skedros et al. Adapted with permission from John Wiley and Sons. Copyright 2005^20^. **d** The intertrabecular spaces in antler bone are occupied by primary osteons. Trabeculae (asterisks) and unremodelled islands of calcified cartilage (arrow) can be identified. From Kierdorf et al. Adapted with permission from John Wiley and Sons. Copyright 2013^30^. **e** Topographical BSE-SEM. For each 90° sector of an annular BSE detector, a separate image is recorded containing information sensitive to the direction of apparent illumination (arrows). From Boyde A. Adapted with permission from John Wiley and Sons. Copyright 2003^31^. **f** Osteon pull-out under cyclic mechanical loading observed using SE imaging. From Hiller et al. Adapted with permission from John Wiley and Sons. Copyright 2003^47^. **g** Cell surface detail of osteoblasts on the surface of parietal bone. From Jones S. J. Adapted with permission from Springer Nature. Copyright 1974^57^. **h** Osteoblasts appear to organise collagen fibrils through flat basal processes. From Pazzaglia U.E. et al. Adapted with permission from Springer Nature. Copyright 2010^61^. **i** Hypermineralised osteocyte lacuna containing mineralised apoptotic debris. From Shah et al. Adapted with permission from the American Chemical Society. Copyright 2017.^69^ **j** Severely disorganised bone microstructure in melorheostosis. From Fratzl-Zelman et al. Adapted with permission from John Wiley and Sons. Copyright 2019^72^. **k** Bone with (right) and without (left) osteocytes. From Atkins et al. Adapted with permission from the National Academy of Sciences. Copyright 2014^77^. **l** An osteocyte and associated canalicular network exposed by resin cast etching. From Feng et al. Adapted with permission from John Wiley and Sons. Copyright 2006^79^. **m** Howship’s lacunae: longitudinally extended resorption (LER; left) and reticulate patch resorption (RPR; right) lacunae. From Gentzsch et al. Adapted with permission from Springer Nature. Copyright 2003^84^. **n** Cryo-SEM. The combination of SE (above) and BSE (below) imaging provides morphological and compositional information. From Mahamid et al. Adapted with permission from the National Academy of Sciences. Copyright 2010^97^

BSE is the most useful operating mode for compositional imaging of bone, allowing for discrimination between mineralised and unmineralised compartments (Fig. 2). Cortical porosity varies with respect to gender,^7^ as a function of age,^8,9^ increases in experimentally induced osteoporosis,^10^ and decreases with anti-resorptive treatment.^11^ The osteocyte lacuno-canalicular network also contributes to the overall porosity. Reportedly, osteocyte lacunar density differs between circumferential (periosteal and endosteal) lamellar areas and central areas in rat femoral cortical bone.^12^ Variations in mineral density and rate of bone turnover/remodelling can be readily probed.^13,14^ At a typical osteotomy site, woven bone appears less homogenously mineralised than pre-osteotomised lamellar bone.^15^ Disordered collagen fibrils laid down initially undergo mineralisation and fusion into bundles of mineralised collagen fibrils, before being gradually replaced by ordered mineralised tissue.^16^ During remodelling, osteoclasts may perforate trabeculae and disrupt their structure. Such damage is typically repaired by means of a *bridge* of lamellar bone deposited in a specified direction.^17^ Trabecular repair may occasionally occur via a *microcallus* whereby a globular woven bone formation transiently reconnects two (or more) elements.^18^ The healing pattern is, however, influenced by the surgical technique employed for osteotomy preparation. Drilling with conventional steel burs generates bone fragments while piezosurgery and laser ablation, both, produce clean and smooth walls that lead to more advanced initial healing.^19^ The boundaries between secondary osteons and interstitial bone, and between individual trabecular packets are formed by cement lines, which are relatively hypermineralised in comparison and therefore appear brighter.^20,21^ Unremodelled islands of mineralised cartilage can also be detected,^22,23^ without the need for specific staining procedures. In the human jaw, regions of high mineralisation density correspond to sites that are predicted to experience the highest principal strains during biting.^24^ Disease conditions affecting bone mineralisation can be easily identified using BSE-SEM. In osteopetrosis, the presence of sclerosis is noted with variations in degrees of lamellar bone mineralisation and partial obliteration of bone marrow cavities.^25^ Osteomalacia manifests as nearly complete failure of mineralisation in the bone surrounding blood vessel canals and arrested mineralisation fronts characterised by a failure of fusion of calcospherulite-like micro-volumes within bone.^26^ Bone obtained from an atypical femoral fracture associated with long-term anti-resorptive use shows highly mineralised, porous tissue containing many enlarged osteocyte lacunae, on to which lamellar bone is formed.^27^ In the case of prematurely fused cranial sutures, osteonal features such as cement lines are visible and the outlines of mineralised sutures are smooth. In comparison, patent suture margins show large amounts of woven bone and disrupted mineralisation fronts.^28^

Antler bone ranks among the toughest biological materials and is subjected to high impact loading and large bending moments.^29^ The microstructure of the antler cortex, for instance in the European roe deer (*Capreolus capreolus*), comprises largely of a network of trabecular bone of endochondral origin. Intertrabecular voids are later filled in by primary osteons—a process that is apparently preceded by bone resorption on the trabecular surface, as interpreted from the occurrence of cement lines around primary osteons.^30^

A ‘topographical BSE’ approach has also been explored for obtaining directional information from complex 3D shapes such as trabecular bone. A separate image is recorded for each 90° sector of an annular BSE detector. The information contained in each image is sensitive to the direction of apparent illumination. An extended depth of field can be attained by collecting a series of images while physically moving the sample towards the detector.^31^

SE imaging is most suitable for imaging of surfaces. It has long been recognised that bone surface morphology reflects the local metabolic activity of bone cells.^32^ The orientation of collagen tends to be the same as the osteoblast that has produced it.^33^ Collagen organisation and the appearance of resorption pits, however, vary as a function of age.^34^ Age-related changes in bone architecture can be easily studied using SEM.^35^ Bone formation and bone resorption activities display morphological coupling in younger individuals and uncoupling in elderly individuals.^36^ In cortical bone, osteon development is discontinuous with variable lamellar apposition rates and Haversian canal circumference reduction in the direction opposite to the advancing cutting cone.^37^ Failure surfaces arising from monotonic fractures,^38^ accumulation of fatigue microdamage,^39^ experimentally induced fractures,^40^ tensile testing,^41,42^ three-point bending,^43,44^ and crack propagation testing using notched specimens^45^ can also be investigated. Bone behaves like a tough material at low strain rates exhibiting “pull-out” failure, but fractures like a brittle material at high strain rates exhibiting a tensile failure pattern.^46^ Loading conditions (e.g., monotonic or fatigue failure) and the local microstructure influence the extent of osteon pull-out, which is considered an important toughening mechanism in cortical bone.^47^ Other toughening mechanisms (e.g., uncracked ligaments and crack deflections) are anisotropic and vary with respect to the direction of crack propagation, i.e., longitudinal, radial, and transverse.^48^ Most interestingly, presence is suggested of a non-fibrillar organic matrix component that holds mineralised collagen fibrils together and resists their separation.^49^

With particular reference to crack propagation in bone, the anisotropic toughness behaviour is a direct consequence of the various extrinsic toughening mechanisms associated with specific microstructural features.^50^ Similarly, under shear loading the largest proportion of cracks reaching an osteon propagate into the osteon for a few lamellae before being deflected by the lamellar structure in a circular path and exit the osteon. Often cracks pass through the central Haversian canal without being deflected. On occasion, crack deflection is also noted along cement lines.^51^

The structural role of water in osteonal lamellar bone has been explored through in situ dehydration–rehydration experiments. The loss of bulk and weakly bound water leads to 1.2%–1.4% contraction, which has been attributed to the presence of more water-containing rather than mineral-containing spaces within mineralised collagen fibril arrays.^52^ However, even in the most potentially dehydrating environment inside the SEM, elastic modulus values of bone remain independent of vacuum conditions (tested up to 5.25 × 10^−4^ Pa pressure and 2 h exposure time).^53^

Interfaces between bone and fibrous connective tissues (e.g., ligaments and tendons) are highly interesting from a biomechanical point of view. The bone–ligament interface exhibits a sharp transition in the mineral content where “fingers” of mineralised matrix surround hypertrophic chondrocyte lacunae.^54^ The bone–tendon junction is characterised by an intertwined network of collagen fibrils surrounding lacunae of fibrocartilage cells and lipid droplets among collagen fibres of the tendon.^55^

For detailed descriptions of specific sample preparation protocols, the interested reader is referred elsewhere.^56^

### Osteoblasts and osteocytes

Both SE and BSE modes allow easy access to osteoblasts and osteocytes. On the surface of rat parietal bone, the secretory territory of osteoblasts is estimated at 154 µm^2^ per cell.^57^ The various phenotypic stages in the transformation of matrix-producing cells at the bone surface to terminally differentiated, mineralised matrix-bound osteocytes have been studied by exposing bone specimens to different chemicals, e.g., collagenase,^58^ osmium tetroxide (OsO_4_) and potassium ferrocyanide [K_4_Fe(CN)_6_],^59^ sodium hypochlorite (NaOCl),^60^ etc. Prolonged immersion in OsO_4_ (48 h–72 h) removes most of the osteoblast cell body, exposing flat, finger-like basal projections of osteoblasts that seemingly arrange collagen fibrils into compact, ordered bundles.^61^ Approximately one in every 67 osteoblasts terminally differentiates into an osteocyte.^59^ It is now understood that osteocytes play a key role in the calcium metabolism, as exemplified by the enlargement of osteocyte lacunae during periods of high calcium demands such as lactation.^62,63^ The spatial distribution of osteocytes may be affected in certain conditions. An example is of prematurely fused cranial sutures where osteocytes appear better organised and are systematically interconnected via canaliculi in contrast to patent sutures where osteocytes tend to be more disorganised.^28^

Investigated in the medial, lateral, dorsal, and plantar cortices of calcanei in elk, horse, and sheep, reportedly site-specific and inter-species variations in osteocyte density correlate poorly with local structural and material properties, such as strain distribution patterns.^64^ In humans, the proportion of hypermineralised osteocyte lacunae increases with age.^65^ Such osteocyte lacunae contain mineralised apoptotic debris, and can be detected in a variety of circumstances, e.g., lacunae adjacent to a metal implant stem in the human femoral shaft,^66^ in patients aged 2–23 years diagnosed with osteogenesis imperfecta (types I, III, IV, V),^67^ in osteoporotic and osteoarthritic human trabecular bone,^68^ in bisphosphonate-exposed human alveolar bone where facetted crystals of magnesium whitlockite were identified,^69^ in human auditory ossicles,^70^ and in archaeological bone.^71^ In some disease conditions, e.g., melorheostosis, intense bone formation activity is linked with entrapment of osteocytes in greater numbers (compared to unaffected bone) and therefore higher osteocyte lacunar porosity.^72^ Osteocyte density and morphology also vary between peri-implant bone and native bone with significant implications for bone quality and kinetics of the bone-healing process.^73–76^ Equally intriguing is the complete absence of osteocytes in certain organisms, e.g., in some species of billfish, where there is evidence of bone remodelling despite the absence of strain-sensing capability of osteocytes.^77^

Resin cast etching of bone allows direct visualisation of the osteocyte lacuno-canalicular network.^78^ This technique has revealed that in the absence of dentin matrix protein 1 (DMP1) as in *Dmp1*-deficient mice, the inner lacuno-canalicular wall appears buckled and enlarged.^79^ Furthermore, in DMP1 and *Klotho* deficient (*Dmp1*^*–/–*^ *kl/kl*) mice, osteocytes are poorly organised, visibly larger in size, and exhibit a complete lack of cell processes.^80^ Similarly, disruption of the von Hippel–Lindau gene (*Vhl*) activates the HIFα signalling pathway, which results in osteocytes that appear disorganised, randomly oriented, irregularly contoured, and fewer in number.^81^ Osteocytes also exhibit morphological and structural abnormalities in unloaded and/or ovariectomised conditions, which may be reversed by blockade of protein sclerostin through administration of sclerostin antibodies.^82^ Direct attachment of osteocytes to the surface of implanted biomaterials can also be evaluated,^74,83^ and may be interpreted as an indication of osseointegration when observed at materials otherwise considered to integrate poorly, e.g., CoCr.^73,75^

### Howship’s lacunae

Resorption lacunae, or Howship’s lacunae, on the surface of trabecular bone can be visualised after brief deproteinisation.^36,60^ Here, two morphologically distinct types of resorption lacunae exist: (i) longitudinally extended resorption lacunae (LER), and (ii) reticulate patch resorption lacunae (RPR).^84,85^ Thought to represent different stages in the resorption process, two further types of resorption lacunae have been described depending on the appearance of the lacunar surface: (i) rough (*type-I*), due to the presence of loose collagen fibrils, and (ii) smooth (*type-II)*, with almost no fibrillar structures.^86^ A stereoscopic imaging (3D SEM) approach has also been proposed to quantitatively analyse the topography of osteoclastic excavations on slices of devitalised cortical bone.^87^

### Bone mineral density distribution

The intensity of BSEs is proportional to the concentration of bone mineral (in wt% Ca). Quantitative backscattered electron imaging (qBEI) is a technique by which bone mineral density distribution (BMDD) may be determined. BMDD reflects bone turnover, mineralisation kinetics, and the average tissue age. The BSE signal is calibrated using stoichiometric hydroxyapatite (HAp), carbon (*Z* = 6) and aluminium (*Z* = 13), and/or other reference standards of known average atomic number.^88,89^ Some outstanding examples of qBEI use include assessment of BMDD in type-2 diabetes,^90^ osteoporosis,^91^ osteoarthritis,^68^osteogenesis imperfecta type I,^92^ osteogenesis imperfecta type VI,^93^ bisphosphonate treatment,^94^ etc. Furthermore, BMDD measurements have revealed that osteoid volume is the main predictor of mineralisation heterogeneity,^95^ and defects of the COLIa1 gene negatively affect bone mineralisation.^96^

### Cryo-SEM

Rapid high-pressure freezing enables preservation of tissue in near-native/hydrated state. BSE and SE imaging are performed at low temperatures (e.g., –120 °C) that are achieved with liquid nitrogen. Cryo-SEM thus provides compositional and topographical information without introducing artefacts associated with drying. A striking example is of mineral-bearing globular deposits in newly mineralised and platelet-like mineral particles in mature mineralised bone in zebrafish caudal fins.^97^ Similar mineral-bearing globular deposits have been reported within cells lining the forming surfaces of mouse femur and calvarium. Contained within 1 µm diameter vesicles, intracellular mineral is aggregated into 80 nm diameter globules that are frequently interconnected by fibrillar structures.^98^ Intracellular membrane-bound mineral deposits are also found in rapidly forming long bones of the chicken embryo.^99^

## Bone around implant biomaterials

In the context of bone regeneration around implant biomaterials, where commercially pure titanium (cp-Ti)^100^ and titanium alloys (TI6Al4V)^101,102^ are typical examples, adhesion of bone-like tissue to the implant surface can be observed directly (i.e., without prior resin embedding) using SE imaging. Interestingly, the orientation of collagen in the first layers interfacing the implant is strongly influenced by the microtexture of the implant surface.^103^ BSE imaging, however, enables characterisation of bone microstructure in much more detail, for example to distinguish between newly formed woven bone and remodelled lamellar bone, or between the two major bone types, i.e., cortical and trabecular. Moreover, finer details such as the density, shape, and size of osteocyte lacunae, as well as the area fraction occupied by osteocyte lacunae and blood vessels and therefore the porosity can also be measured. Newly remodelled areas as well as sites of ongoing osteoclastic activity can be identified. The bone surface is identified by the presence of osteoblastic-osteocytes (partially embedded osteocytes) close to the mineralisation front, which is granular in appearance.

**Fig. 3:**
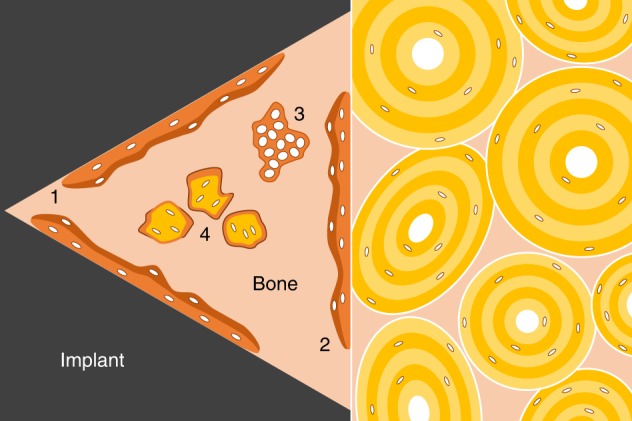
Sources of osteogenesis around implant biomaterials. 1. Contact osteogenesis. 2. Distance osteogenesis. 3. De novo formed woven bone. 4. Autogenous bone fragments

Of much relevance to understanding the osteogenic potential of a given set of implant design features, it is vital to distinguish between the various sources of bone formation within the healing defect. Bone formation process that begins at the implant surface, i.e., in response to the physico-chemical properties of the implant surface, is referred to as *contact osteogenesis*.^104^ Bone also forms in order to occupy the remaining space, to which several processes contribute. For instance, bone that forms on the bony margin of the surgical defect, or referred to as *distance osteogenesis*,^104^ and de novo formed woven bone (Fig. 3). The healing patterns differ between critical and sub-critical sized defects.^105^ In critical sized defects, woven bone forms as a ‘first wave’ of osteogenesis and is later remodelled and replaced by more ordered lamellar bone.^106^ Indeed, the same is true in the case of rapidly growing bone, where poorly ordered bone behaves as a natural *scaffold* for the formation of ordered lamellar tissue.^107^ Frequently, autogenous bone fragments (or bone chips) originating from surgical drilling may be identified in the early stages of healing, and are able to support bone formation directly on their surface.^108–110^

Percentage bone-implant contact and bone area (also referred to as *bone volume* and *bone density*) are important quantitative measures of osseointegration. These are easily assessed using stained histological sections. Bone area measurements made using BSE-SEM are generally comparable with histology^111^ and X-ray micro-computed tomography.^112^ However, histological sectioning may introduce height separation artefacts at the bone-implant interface, thereby precluding accurate determination of bone-implant contact.^113^ Such artefacts are encountered less frequently with BSE-SEM.^73^

**Fig. 4:**
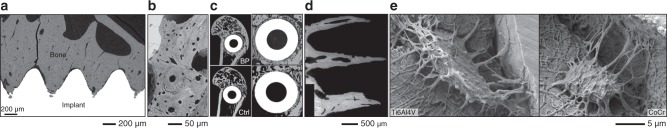
Imaging bone around implant biomaterials. **a** BSE imaging of bone formed around laser-ablated titanium. From Palmquist et al. Adapted with permission from John Wiley and Sons. Copyright 2011^115^. **b** BSE imaging of HAp-coated titanium implants. From Merolli et al. Adapted by permission from Springer Nature. Copyright 2000^132^. **c** Local bisphosphonate delivery (BP; 16 µg/implant. Ctrl; 0 µg/implant) from HAp-coated cp-Ti implants (3 mm diameter) promotes bone formation. From Peter et al. Adapted with permission from John Wiley and Sons. Copyright 2006^135^. **d** Ingrowth of mineralised tissue into 3D printed polycaprolactone + β-tricalcium phosphate (80:20) scaffolds with a repeating 0°/90° strut laydown pattern. From Paris et al. Adapted with permission from Elsevier. Copyright 2017^153^. **e** Resin cast etching for direct visualisation of osteocyte attachment to various implant surfaces. Example #1: Ti6Al4V. From Shah et al. Adapted with permission from Elsevier. Copyright 2016^74^. Example #2: CoCr. From Shah et al. Adapted with permission from John Wiley and Sons. Copyright 2018^73^

Around metal implants having widely diverging surface and bulk properties, bone formation is routinely examined using BSE imaging (Fig. 4). In addition to machined cp-Ti implants, anodically oxidised cp-Ti,^114^ laser ablated cp-Ti,^115^ and Ti6Al4V,^116^ mesoporous cp-Ti,^117^ micro-porous cp-Ti,^118^ and Ti–Ta–Nb–Zr alloy^119^ exemplify chemical and physical diversity of titanium-based implant biomaterials. BSE imaging is also applicable for ex vivo evaluation of human implants retrieved after long-term clinical function, where characteristic features of successful osseointegration comprise of remodelled lamellar bone and the presence of osteocyte lacunae and associated canaliculi within a few micrometres from the implant surface.^120^

Problems associated with projection effects seen in 2D radiography are absent in BSE imaging,^121^ making the latter advantageous for assessment of bone ingrowth into complex implant designs. Examples of such applications include ex vivo evaluation of porous Ta,^122^ cp-Ti,^123^ Ti6Al4V,^124^ and CoCr.^75^ In addition to comparing implants of different pore dimensions,^125^ the impact of cyclic mechanical loading on bone ingrowth into such geometries has also been investigated.^126^ Addressing the question of the optimum pore size for bone ingrowth, the presence of secondary osteons have been reported within spaces under 75 µm in diameter.^127^

Microscopic fragments of mineralised tissue may remain attached to the implant surface in certain situations. These may include assessment of mechanical anchorage via removal torque^83,116^ and tensile testing,^128^ where such fragments are seen as evidence of excellent interlocking of bone to the implant surface, or when the implant is manually retrieved in order to access implant–adherent cells and/or peri-implant bone for gene expression analysis.^100,129^

In contrast to metal implants, the boundary between bone and degradable implant material migrates over time and advances into the implant giving rise to a characteristic interlocking pattern, as has been demonstrated for HAp + polyhydroxybutyrate composite using BSE imaging^130^ Incorporation of bioactive components, e.g., HAp and calcium silicate, to polyether ether ketone (PEEK) can enhance the bone-bonding behaviour (observed as direct bone-implant contact) of this polymer which otherwise elicits little tissue response and/or bone bonding.^131^ The biological response to metal implants may be enhanced by the application of HAp coatings, eventually giving rise to tightly interlocked lamellar bone with osteocytes in close apposition to the coating.^132^

In osteoporotic conditions, the bone response to local bisphosphonate delivery from HAp-coated cp-Ti implants has been characterised using BSE imaging.^133^ Measurement of bone density gradients with respect to bisphosphonate release, from similar implants,^134,135^ have enabled developing a predictive model of bisphosphonate-loading to maximise peri-implant bone density.^136^ Local bisphosphonate delivery from calcium-deficient apatite granules has been shown to increase the trabecular thickness and bone area in osteoporotic conditions.^137^

In HAp materials, even fissure-like spaces on the order of (1–2) µm (and therefore significantly narrower than the expected dimensions of osteoblasts) can be filled with newly formed bone.^138^ Several other CaP phases, e.g., *α*-tricalcium phosphate^139^ and β-tricalcium phosphate^140^ are known to be osteoconductive. The potential to induce osteogenesis at an ectopic site (i.e., osteoinduction) has been demonstrated for biphasic CaP (HAp + β-tricalcium phosphate) alone,^141^ and as a composite with fibrin glue.^142^ The latter also supports bone regeneration within critical-sized defects in bone.^143^ Establishment of direct interlocking with bone, detectable using BSE imaging, is a feature common to many CaP-based biomaterials.^137–143^

Bone response to various silicate-based bioactive glass and glass-ceramic compositions, e.g., SiO_2_–CaO–Na_2_O–P_2_O_5_,^144^ SiO_2_–CaO–K_2_O–Na_2_O–P_2_O_5_,^144^ SiO_2_–Al_2_O_3_–P_2_O_5_–CaO–CaF_2_,^145,146^ SiO_2_–CaO–K_2_O–MgO–Na_2_O–P_2_O_5_,^147,148^ produced as monoliths and porous scaffolds through a variety of production routes including powder sintering,^144^ lost-wax casting,^145^ selective laser sintering,^146^ unidirectional freezing,^147^ and robocasting^148^ have been investigated using BSE imaging.

Implant materials derived from aragonite (CaCO_3_), sourced from natural coral, and pearl mussel and pearl oyster shells have also been explored for bone repair applications. Using SE and BSE imaging, natural derived CaCO_3_ has been shown to exhibit bone bonding mediated by groups of osteogenic cells that produce mineralising globules and collagen directly at the implant surface,^149^ direct bone-implant contact without intervening soft tissue.^150^ Attributable to erosion, in vivo, the immediate bone-implant boundary displays a toothed-comb appearance.^151^

3D printed polycaprolactone scaffolds incorporating 20% β-tricalcium phosphate and having a repeating 0°/90° strut layout pattern^152,153^ have been used to understand the combined effects of scaffold design, i.e., physical cue, and a range of biological cues on bone regeneration. The latter include bone marrow stromal cells,^154^ recombinant human bone morphogenetic protein-7,^155^ and mesenchymal stem cells,^156^ where BSE imaging has been employed as part of a multiscale analytical toolbox for characterising engineered bone and soft–hard tissue interface.

Resin cast etching is also applicable to bone-implant specimens for direct visualisation of osteocyte attachment to the implant surfaces. Typically in lamellar bone, osteocytes are aligned with their long axes parallel to the surface of cp-Ti implants while their canaliculi may become closely interdigitated with the topographical features^157^, and form an extensive, interconnected lacuno-canalicular system.^158^ Such is also observed adjacent to retrieved clinical dental implants, where osteocytes closest to the implant surface are aligned parallel to the micro-scale contour of the implant surface.^159^ Osteocyte attachment to macro-porous Ti6Al4V and CoCr alloys has also been reported.^74,75^ Degradable materials (e.g., bioactive glass) also support osteocyte attachment via dendritic processes.^160^

## Elemental analysis in the SEM

**Fig. 5:**
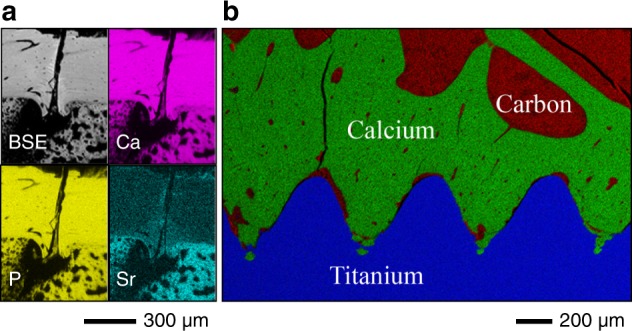
Elemental analysis. **a** BSE image and Ca (magenta), P (yellow), and Sr (cyan) elemental maps demonstrate Sr incorporation into the fracture callus after therapeutic administration. From Brüel et al. Adapted with permission from Springer Nature. Copyright 2011^166^. **b** Colour-merge image C (red), Ca (green), and Ti (blue) elemental maps reveals highly mineralised bone around laser-ablated titanium implants. From Palmquist et al. Adapted with permission from John Wiley and Sons. Copyright 2011^115^

Energy-dispersive X-ray spectroscopy (EDX) uses the X-ray spectrum emitted by a sample when bombarded with a beam of sufficiently energetic electrons to obtain site-specific chemical analysis. A core hole is created when an atom in the sample is ionised by the primary electron beam. An electron from an outer shell transitions into the core hole, generating a characteristic X-ray. EDX can be performed in the SEM using bulk samples with minimal sample preparation. In bone, the most frequent application of EDX is measurement of extracellular matrix Ca and P content (and the Ca/P ratio)^161–163^ (Fig. 5). Across the bone–cartilage interface, Ca levels have been shown to correlate with local nanomechanical properties.^164^ Other examples include detection of intracellular Mg in apoptotic osteocytes,^69,165^ and incorporation of therapeutic elements such as Sr.^166^ Detection of Ca and P along the bone-implant interface confirms the formation of new bone in direct contact with the implant surface,^115,167,168,100^ while presence of Ca and P within topographical features on an implant surface is taken as evidence of bone ingrowth into such features.^169^

## Selective removal and/or preservation of specific tissue components

**Fig. 6:**
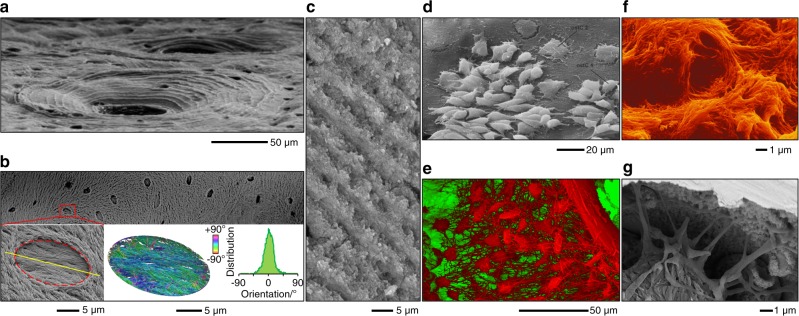
Selective removal and/or preservation of specific tissue components. **a** Osteonal lamellar pattern enhanced by etching with citric acid. From Congiu and Pazzaglia. Adapted with permission from John Wiley and Sons. Copyright 2011.^176^ **b** Osteoblastic-osteocyte lacunae on the surface of trabecular bone treated with NaOCl. From Shah et al. Adapted with permission from Springer Nature. Copyright 2016.^60^ **c** Heat-deproteinised and fractured surface. Ordered layout of mineral crystal aggregates arranged in a concentric sequence of crests and grooves. From Pazzaglia et al. Adapted with permission from John Wiley and Sons. Copyright 2016.^180^ **d** After OsO_4_ and K_4_Fe(CN)_6_ treatment, a portion of lining cells is detached by ultrasonication and the underlying surface is exposed. Two morphological types of cells are recognised here: (i) convex dome-shaped cells with a non-adhering border (denoted as “ostC1”; mean surface area of 52.5 µm^2^ per cell), and (ii) flattened cells on the bone surface with spreading equatorial, cytoplasmic processes (denoted as “ostC2”; mean surface area of 179 µm^2^ per cell). From Pazzaglia et al. Adapted with permission from John Wiley and Sons. Copyright 2014.^59^ **e** Multi-layered cast of the osteocyte network and vasculature obtained by prolonged, repeated exposure to HCl and KOH solutions. From Pazzaglia and Congiu. Adapted with permission from John Wiley and Sons. Copyright 2013.^184^ **f** Directly opposing an implant surface, collagen is exposed by etching with HCl after mechanically separating the implant from a resin embedded bone-implant specimen. From Traini et al. Adapted with permission from John Wiley and Sons. Copyright 2005.^103^ **g** Resin cast etching reveals osteocyte attachment to the surface of a laser-ablated cp-Ti implant. Fine topographical features of the implant surface remain intact after H_3_PO_4_ and NaOCl exposure. From Shah et al. Adapted with permission from the American Chemical Society. Copyright 2015.^159^

Selectively removing (or preserving) certain tissue components is a valuable approach for understanding the contribution(s) of individual components to the overall functional capacity (Fig. 6). For instance, under quasi-static compressive testing, deproteinised trabecular bone (inorganic phase-only) undergoes brittle failure while demineralised trabecular bone (organic phase-only) exhibits ductile failure.^170^ For the purpose of enhancing the contrast of structures, such as reversal lines and interlamellar lines in SE imaging, Congiu et al. presented a thorough appraisal of various reagents including hydrochloric acid (HCl), citric acid (C_6_H_8_0_7_), acetic acid (C_2_H_4_O_2_), sodium phosphate (Na_3_PO_4_), sodium hydroxide (NaOH), and potassium hydroxide (KOH).^171^ While all acidic and alkaline media produce an erosive effect, strong acids and bases are difficult to control. Many different protocols are found in the published literature (Table 1) for exposing and/or enhancing specific components of bone, e.g., the organic phase^172–176^, the inorganic phase,^26,60,86,172,177–181^ cellular content,^58,59,61,78,79,173,182–184^ and the bone-implant interface.^73–75,83,103,157–160,185–189^

**Table 1:** Sample preparation protocols for selective removal and/or preservation of specific tissue components

Application	Reagents, time, temperature	Ref
*Organic phase*		
Surface of trabecular bone	0.6 N HCl, 6 days	^172^
Twisted plywood structure of collagen in lamellar bone	10% EDTA, 3 weeks	^173^
	10% NaOH, 3 days	
	RT	
Widths of successive osteonal lamellae	Resin embedding	^174^
	10% EDTA + 11,190 U·mg^–1^ Trypsin (2:1 mixture), 2 h, 37 °C	
Lamellar pattern and interlamellar lines in osteonal bone	6% Na_3_PO_4_, 1 min, RT	^175, 176^
Lamellar pattern in osteonal bone	6% citric acid, 2 min	^176^
*Inorganic phase*		
Surface of trabecular bone	2.6% NaOCl, 14 days	^172^
Howship’s lacunae	5% NaOCl, 1 min	^86^
Endosteal surface	Tergazyme^TM^, 50 °C	^26^
	5% H_2_O_2_, RT	
Osteoblastic-osteocyte lacunae	5% NaOCl, 3 days	^60^
Stages of mineral maturation; ACP to crystalline CaP	1% NaOCl, <1 min	^177^
Calcospherulites at the mineralisation front	Collagenase (type I) in HBSS (0.5 mg·mL^–1^) + 1 mmol·L^−1^ SPI, 16 h, 37 °C	^178, 179^
Mineral arrangement in osteonal lamellae	500 °C, overnight	^180^
Thermally induced changes in mineral crystal morphology	(200–1 600) °C, (2–24) h	^181^
*Cellular content*		
Basal processes of osteoblasts and exocytosis vesicles	Vascular perfusion (2% FA)	^61^
	1% OsO_4_ + 1.25% K_4_Fe(CN)_6,_ 2 h	
	0.1% OsO_4_, (48–72) h	
Osteoblast–osteocyte transformation	0.25% Collagenase (type I), (0.5–2) h	^58^
Endosteal osteoblast density and osteoblast entrapment index	1% OsO_4_ + 1.25% K_4_Fe(CN)_6,_ 2 h + 6 h	^59^
	Ultrasonication, 30 kHz, 30 s	
Osteocyte network in trabecular bone	6 N HCl, 1 h, 60 °C	^182^
	Collagenase (type II) in 0.1 mol·L^–1^ in PB (1 mg·mL^–1^), 12 h, 37 °C	
Osteocytes, osteoblasts, osteoclasts in parietal bone	4% EDTA + 3.5% sucrose, 16 h	^183^
	5 N KOH, 8 min, 60 °C	
Osteocytes and their processes	10% EDTA, 3 weeks	^173^
	2% Tannic acid, 2 days	
	24% NaOH, 60 °C, 15 min	
Osteocyte network in cortical bone	40% H_2_O_2_, 30 days	^184^
	15% HCl, 15% KOH, alternately, 60 days	
Osteocyte lacuno-canalicular network	Resin embedding	^78, 79^
	9%–37% H_3_PO_4_, (10–30) s	
	5% NaOCl, 5 min	
*Bone-implant interface*		
Bone surface directly opposing the implant surface	Resin embedding	^103^
	0.1 N HCl, 90 s	
	Trypsin (80 U·mL^–1^), 41 h, 37 °C	
Osteocyte attachment to implant biomaterials^a^	Resin embedding	^73– 75, 83, 157– 160, 185, 186^
	9%–37% H_3_PO_4_, (10–30) s	
	5% NaOCl, 5 min	
Osteocytes, osteoblasts, osteoclasts around HAp implants	10% EDTA + 3.5% sucrose, 3 days	^187^
	5 N KOH, 8 min, 60 °C	
Bone cells, collagen fibres, blood vessels around HAp implants	Vascular perfusion (RS, 2.5% GA, MA)	^188^
	N_2_ freeze-fracture	
Angiogenesis within degrading HAp implants	Vascular perfusion (RS, 1% GA, MA)	^189^
	10% HNO_3_, 12 h	
	20% KOH, 20 h	

Tergazyme^TM^, alkaline bacterial pronase enzyme detergent; SPI, serine protease inhibitor, 4-(2-Aminoethyl)benzenesulfonyl fluoride hydrochloride; RT room temperature; PB, phosphate buffer; RS, Ringer’s solution; FA, formaldehyde; GA, glutaraldehyde (phosphate buffered); MA, methacrylate resin

^a^Examples include bioactive glass, microporous β-TCP, machined and surface modified cp-Ti, CoCr, macro-porous alloys

## SEM in paleoarchaeology

**Fig. 7:**
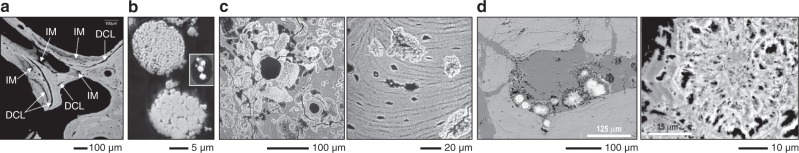
SEM in paleoarchaeology. **a** Defects of active osteomalacia visible in archaeological bone from the adult rib. Multiple areas of incomplete mineralisation (IM) and defect cement lines (DCL) are noted. From Brickley et al. Adapted with permission from John Wiley and Sons. Copyright 2007.^195^ **b** Pyrite deposits within Haversian canals in human tibia from 2 000 years ago. From Tjelldén et al. Adapted with permission from John Wiley and Sons. Copyright 2018.^202^ **c** A number of high-density foci within a single secondary osteonal system in archaeological human tibia (left). Bacterial ingress seen extending from a single osteocyte lacuna. Other osteocytes exhibit demineralisation boundaries or enlargement (right). From Bell LS. 2012. Forensic Microscopy for Skeletal Tissues. Adapted with permission from Springer Nature. Copyright 2012.^196^ **d** Mycelia mineralised with Fe/Mn oxides and calcite. Here, the mycelia (white) are seen as sunflower-like aggregates and networks of hyphae filling the resorption canals inside the compact bone tissue. From Owocki et al. Reproduced under the terms of the Creative Commons Attribution License (CC BY 4.0)^204^

The SEM is a key analytical tool in archaeological science (Fig. 7). Using a two-step technique for replicating the specimen surface, mapping of bone-remodelling patterns and growth dynamics has been able to explain the apomorphic features of the Neanderthal mandible.^190^ Morphological features peculiar to various diseases including syphilis,^191,192^ infantile scurvy,^193^ rickets,^194^ osteomalacia,^195^ etc. have been noted in archaeological human bone. Moreover, post-mortem changes in bone,^196^ taphonomic processes,^197,198^ the extent of bioerosion,^199^ bacterial and fungal attack on fossil bone^200^ may also be thoroughly characterised.

Detection of mineral inclusions such as manganese oxide,^201^ pyrite,^202^ calcite,^203^ etc. reveal vital clues pertaining to the chemical and physical environments that the samples had been exposed to. For example, the presence of ferromanganese oxides filling diagenetic cracks in dinosaur bone points towards a fungal activity-mediated process of decay.^204^ Alternatively, identification of osteocyte-like structures in bones from mastodon,^205^ and several dinosaur species including Brachylophosaurus,^206^ Tyrannosaurus,^207^ and Triceratops^208^ provides useful estimate of the quality of tissue preservation.

## FIB-SEM for 3D imaging and sample preparation

**Fig. 8:**
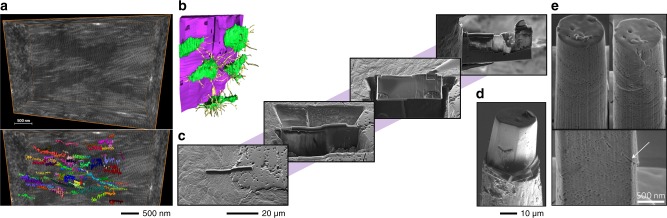
Focused ion beam (FIB) techniques for 3D imaging and sample preparation. **a** FIB tomography of collagen fibrils in bone. From Reznikov et al. Adapted with permission from the American Association for the Advancement of Science. Copyright 2018.^211^ **b** FIB tomography of implanted demineralised dentin matrix and surrounding new bone where osteocytes form an interconnected network of cellular processes. From Tanoue et al. Reproduced under the terms of the Creative Commons Attribution License (CC BY 4.0).^216^ **c** Sample preparation for transmission electron microscopy using the in situ lift-out technique, starting with deposition of a 30 µm long, 2 µm wide, and 1 µm-thick protective layer, followed by sequential milling, lift-out, and thinning to electron transparency. From Grandfield et al. Adapted with permission from John Wiley and Sons. Copyright 2012.^217^ **d** Sample preparation for ptychographic X-ray computed tomography. From Dierolf et al. Adapted with permission from Springer Nature. Copyright 2010.^229^ **e** Micropillars and nanopillars prepared for uniaxial compression testing. From Tertuliano and Greer. Adapted with permission from Springer Nature. Copyright 2016^231^

In addition to an electron beam, commercial FIB-SEM instruments have an ion beam column. The electron and ion beam columns are oriented between 45° and 55° relative to each other and are capable of being operated independently. When a sample is placed at the eucentric height where the two beams coincide, and tilted so that the sample surface is normal to the ion beam, it is possible to simultaneously image using electrons and mill using ions, typically gallium since it has a low melting point (around 30 °C). The most common applications of FIB-SEM instruments include 3D imaging (or *FIB tomography*) and sample preparation for other analytical techniques (Fig. 8).

Without the need for specific staining procedures, Schneider et al. demonstrate the applicability of FIB tomography for high-resolution morphometry of the osteocyte lacuno-canalicular network, which is recorded as a negative imprint of the surrounding mineralised extracellular matrix.^209^ However, through demineralisation using ethylenediaminetetraacetic acid (EDTA; C_10_H_16_N_2_O_8_) followed by OsO_4_ and K_4_Fe(CN)_6_ staining, cytoplasmic processes at the osteoblast–osteoid interface have been shown to be tubular in appearance.^210^ The ultrastructural arrangement of collagen fibrils can also be investigated using FIB tomography.^211^ In fibrolamellar bone, the arrangement of collagen fibrils is highly anisotropic and mainly oriented parallel to the major loading axis.^212^ The collagen fibril arrangement in human cortical bone appears to be such that each lamella is composed of ordered and disordered regions, with the network of canaliculi restricted to the latter.^213^ FIB tomography also reveals that the strong anchorage of periosteum to bone is a result of collagen fibre bundles perforating the bone surface at ~30° angles and forming a net-like structure.^214^ Ingrowth of mineralised bone into volcano-like, 250 nm to 2 µm, topographical features of a titanium implant has also been visualised by 3D reconstruction of 100 nm-thick serial slices.^215^ Likewise, the attachment of osteocytes via cell processes to demineralised dentin matrix particles implanted in the rat calvarium has also been studied using FIB tomography.^216^

FIB-SEM facilitates preparation of samples for various analytical techniques, many of which pose highly exacting geometrical requirements. A prime example is of transmission electron microscopy (TEM) where sample thickness must be ~100 nm (or better) in order to achieve electron transparency. Several analytical options are available in the TEM, for instance high-angle annular dark field scanning transmission electron microscopy for compositional (*Z*-) contrast,^112,217–219^ high-resolution transmission electron microscopy for direct imaging of the atomic structure,^220–222^ electron diffraction for studying the crystal structure,^69^ electron tomography for 3D imaging,^223,224^ and electron energy-loss spectroscopy^69,159^ and EDX for elemental analysis.^220,225^ Other techniques for which samples can be prepared using FIB-SEM include atom probe tomography,^222,226,227^ time-of-flight secondary ion mass spectrometry,^225,228^ ptychographic X-ray computed tomography,^229^ and microscale and nanoscale mechanical testing.^230,231^

## SEM in combination with other analytical techniques

The wide range of applicable sample processing routes and the relatively few geometrical constraints imply that the same sample may be used for multiple analytical methods, either directly or after minor adaptations. Alternatively, for analytical methods that pose specific requirements in terms of sample thickness and/or operate in transmission mode, e.g., optical microscopy, thinner samples can usually be obtained from larger SEM preparations. In some cases, certain characteristics of a given preparation may be undesirable, e.g., heavy metal contrast staining may interfere with X-ray fluorescence and vibrational spectroscopy. Likewise, embedding media may exhibit autofluorescence or could otherwise compromise the validity of acquired data, such as mechanical testing. Nevertheless, the information acquired from SEOsteocyte attachment toM investigations can be easily correlated with other analytical methods. Examples include histology using optical microscopy,^152,153^ polarised light microscopy,^175^ second harmonic generation microscopy,^232,233^ confocal laser scanning microscopy,^234,235^ small-angle X-ray scattering,^15,105,236^ Raman spectroscopy,^69,237^ failure testing,^238^ in situ crack propagation studies,^239–243^ nanoindentation^164,244–247^ including in situ measurements,^248^ and in situ atomic force microscopy.^249–251^

## Limitations, pitfalls, and future outlook

In addition to the many advantages, e.g., very high spatial (*x*–*y* and *z*) resolution, large depth of field, and wide field of view, that attest in favour of the utility of the SEM for studying a compositionally and structurally complex system, such as bone, the instrument is not without certain idiosyncratic limitations and pitfalls. To list a few:

- Bone cells (or intracellular organelles) cannot be observed directly using 2D BSE imaging, except for those that are surrounded by mineral (e.g., osteocytes and osteoblastic-osteocytes). Different contrast staining techniques have been advocated. Examples include the “*OTOTO* protocol” involving sequential application of osmium tetroxide (O; OsO_4_) and thiocarbohydrazide (T; CH_6_N_4_S),^213^ potassium triiodide (Lugol’s solution),^252^ OsO_4_ and K_4_Fe(CN)_6_,^210^ etc. Heavy metal staining procedures employing OsO_4_, uranyl acetate (C_4_H_8_O_6_U), and lead citrate (C_12_H_10_O_14_Pb_3_) have been used to observe cells in peri-implant bone, including osteoblasts and erythrocytes within blood vessels.^163^

For the purpose of mineralised tissue morphometry, although BSE imaging affords greater flexibility in specimen thickness/height compared to optical microscopy using histological sections, certain stains (e.g., Masson’s or Goldner’s trichrome, Movat’s pentachrome) enable discrimination between osteoid and mineralised bone. Together with polarised light, the picrosirius red stain relies on the birefringent properties of collagen molecules to selectively highlight the collagen network in tissues. The rate of bone formation can be quantified using fluorescent labelling with dyes such as calcein and alizarin. Furthermore, tartrate-resistant acid phosphatase (TRAP) staining can be used for identification of osteoclasts. Cell nuclei, not typically observed with BSE imaging, are also easily detected using histology.

The resin cast etching technique does not, in fact, show the osteocytes or their dendritic extensions. What is observed is the embedding resin that has infiltrated into the pericellular space, thereby encapsulating the cellular components. It is, therefore, reasonable to assume that the topography observed on any given structure is effectively a negative imprint of the mineralised surface that it previously opposed.^159^

Microcracks may appear in resin embedded specimens due to poor handling, polishing, drying, etc. Careful consideration must be given to such features where quantification of the unmineralised compartment is desired.

Electron beam-induced damage may alter the mineral content. Apparent increases in the relative proportions of Ca and P, measured using EDX, are likely a result of decreased C content.^253^ For this reason, Ca/P ratios may be more reliable than absolute Ca and P content.

Monte Carlo simulations of electron trajectories suggest that the X-ray generation volume is considerably greater than the volume through which BSEs travel. Additionally, these volumes vary between elements, and therefore Ca/P ratios measured using EDX in the SEM, may be less than accurate.^254^ Such measurements are better carried out using thin samples in a TEM where errors originating from variable interaction volumes are expected to be minimal.

While analytical options such as cathodoluminescence^255^ and electron backscatter diffraction^256^ have been of limited usefulness over the years, the trend towards 3D imaging using FIB-SEM instrumentation has become progressively ubiquitous. Although recent advances in integrated correlative light and electron microscopy have found broad applicability in biology,^257^ there remain opportunities to directly visualise molecular events in bone. More recent developments include high-throughput imaging of macroscopic tissue samples at nanoscale resolution using multi-beam SEM instruments equipped with as many as 61 parallel electron beams.^258^ Further extending the analytical capabilities of the SEM, developments in soft X-ray emission spectroscopy enable high resolution chemical state analysis at par with X-ray photoelectron spectroscopy and electron energy-loss spectroscopy, with high sensitivity for trace element detection (<100 mg·L^–1^).^259^ The last 50 years have witnessed the SEM evolve from a surface imaging tool requiring tedious sample processing into a highly sophisticated, nanoanalytical powerhouse capable of being operated at low accelerating voltages and variable vacuum conditions, with a diverse selection of in situ experimental possibilities to choose from. Some novel uses of the SEM include observation of heat-induced alteration to the mineral phase of human bone, which begins to undergo recrystallisation at 600 °C, resulting in a range of crystal morphologies from spherical, hexagonal, platelets, rosettes. Further heating leads to fusion of crystals at 1 000 °C, and melting at 1 600 °C.^181^ Attempts have also been made to identify characteristic microscopic features of sharp force trauma to bone.^260^ Another potential application is investigation of wildlife crime, where the combination of imaging and chemical analysis in the SEM may be used to ascertain the nature of suspected ballistic fragments in bone.^261^ These unique and diverse applications are a testament to the versatility and user-friendly nature of this particular instrument. Considering the current trends in technological advancement, major developments in the analysis of bone using the SEM can be foreseen in the years to come. The possibilities are endless.

## Supplementary information

Permission to reuse content

Permission to reuse content

Permission to reuse content

Permission to reuse content

Permission to reuse content

Permission to reuse content

Permission to reuse content

Permission to reuse content

Permission to reuse content

Permission to reuse content

Permission to reuse content

Permission to reuse content

Permission to reuse content

Permission to reuse content

Permission to reuse content

Permission to reuse content

Permission to reuse content

Permission to reuse content

Permission to reuse content

Permission to reuse content

Permission to reuse content

Permission to reuse content

Permission to reuse content

Permission to reuse content

Permission to reuse content

Permission to reuse content

Permission to reuse content

Permission to reuse content

Permission to reuse content

Permission to reuse content

Permission to reuse content

Permission to reuse content

Permission to reuse content

Permission to reuse content

Permission to reuse content
