# Supplementary material for: 50 years of scanning electron microscopy of bone—a comprehensive overview of the important discoveries made and insights gained into bone material properties in health, disease, and taphonomy
Source: Bone Res. 2019 May 22;7:15. doi: 10.1038/s41413-019-0053-z (PMC6531483; doi:10.1038/s41413-019-0053-z)
Supplement: Supplementary file 5 — Permission to reuse content [file 41413_2019_53_MOESM5_ESM.pdf]

JOHN WILEY AND SONS LICENSE
TERMS AND CONDITIONS

Mar 02, 2019

This Agreement between Furqan A Shah ("You") and John Wiley and Sons ("John Wiley and Sons") consists of your license details and the terms and conditions provided by John Wiley and Sons and Copyright Clearance Center.

License Number	4541120373513
License date	Mar 02, 2019
Licensed Content Publisher	John Wiley and Sons
Licensed Content Publication	Journal of Anatomy
Licensed Content Title	The cast imaging of the osteon lacunar-canalicular system and the implications with functional models of intracanalicular flow
Licensed Content Author	Terenzio Congiu, Ugo E. Pazzaglia
Licensed Content Date	Oct 22, 2012
Licensed Content Volume	222
Licensed Content Issue	2
Licensed Content Pages	10
Type of use	Journal/Magazine
Requestor type	University/Academic
Is the reuse sponsored by or associated with a pharmaceutical or medical products company?	no
Format	Print and electronic
Portion	Figure/table
Number of figures/tables	1
Original Wiley figure/table number(s)	Figure 6
Will you be translating?	No
Circulation	10000
Title of new article	50 years of scanning electron microscopy of bone – A comprehensive overview of the important discoveries made and insights gained into bone material properties in health, disease, and taphonomy
Publication the new article is in	Bone Research
Publisher of new article	Springer Nature
Author of new article	Furqan A. Shah, Krisztina Ruscsák, Anders Palmquist
Expected publication date of new article	Aug 2019
Estimated size of new article (pages)	12
Requestor Location	Furqan A Shah

Department of Biomaterials
Arvid Wallgrens Backe 20
Box 412
Göteborg, 405 30
Sweden
Attn: Furqan A Shah

Publisher Tax ID	EU826007151
Total	0.00 USD

Terms and Conditions

TERMS AND CONDITIONS

This copyrighted material is owned by or exclusively licensed to John Wiley & Sons, Inc. or one of its group companies (each a"Wiley Company") or handled on behalf of a society with which a Wiley Company has exclusive publishing rights in relation to a particular work (collectively "WILEY"). By clicking "accept" in connection with completing this licensing transaction, you agree that the following terms and conditions apply to this transaction (along with the billing and payment terms and conditions established by the Copyright Clearance Center Inc., ("CCC's Billing and Payment terms and conditions"), at the time that you opened your RightsLink account (these are available at any time at http://myaccount.copyright.com).

Terms and Conditions

- The materials you have requested permission to reproduce or reuse (the "Wiley Materials") are protected by copyright.

- You are hereby granted a personal, non-exclusive, non-sub licensable (on a stand-alone basis), non-transferable, worldwide, limited license to reproduce the Wiley Materials for the purpose specified in the licensing process. This license, **and any CONTENT (PDF or image file) purchased as part of your order,** is for a one-time use only and limited to any maximum distribution number specified in the license. The first instance of republication or reuse granted by this license must be completed within two years of the date of the grant of this license (although copies prepared before the end date may be distributed thereafter). The Wiley Materials shall not be used in any other manner or for any other purpose, beyond what is granted in the license. Permission is granted subject to an appropriate acknowledgement given to the author, title of the material/book/journal and the publisher. You shall also duplicate the copyright notice that appears in the Wiley publication in your use of the Wiley Material. Permission is also granted on the understanding that nowhere in the text is a previously published source acknowledged for all or part of this Wiley Material. Any third party content is expressly excluded from this permission.

- With respect to the Wiley Materials, all rights are reserved. Except as expressly granted by the terms of the license, no part of the Wiley Materials may be copied, modified, adapted (except for minor reformatting required by the new Publication), translated, reproduced, transferred or distributed, in any form or by any means, and no derivative works may be made based on the Wiley Materials without the prior permission of the respective copyright owner.**For STM Signatory Publishers clearing permission under the terms of the STM Permissions Guidelines only, the terms of the license are extended to include subsequent editions and for editions in other languages, provided such editions are for the work as a whole in situ and does not involve the separate exploitation of the permitted figures or extracts,** You may not alter, remove or suppress in any manner any copyright, trademark or other notices displayed by the Wiley Materials. You may not license, rent, sell, loan,

lease, pledge, offer as security, transfer or assign the Wiley Materials on a stand-alone basis, or any of the rights granted to you hereunder to any other person.

- The Wiley Materials and all of the intellectual property rights therein shall at all times remain the exclusive property of John Wiley & Sons Inc, the Wiley Companies, or their respective licensors, and your interest therein is only that of having possession of and the right to reproduce the Wiley Materials pursuant to Section 2 herein during the continuance of this Agreement. You agree that you own no right, title or interest in or to the Wiley Materials or any of the intellectual property rights therein. You shall have no rights hereunder other than the license as provided for above in Section 2. No right, license or interest to any trademark, trade name, service mark or other branding ("Marks") of WILEY or its licensors is granted hereunder, and you agree that you shall not assert any such right, license or interest with respect thereto

- NEITHER WILEY NOR ITS LICENSORS MAKES ANY WARRANTY OR REPRESENTATION OF ANY KIND TO YOU OR ANY THIRD PARTY, EXPRESS, IMPLIED OR STATUTORY, WITH RESPECT TO THE MATERIALS OR THE ACCURACY OF ANY INFORMATION CONTAINED IN THE MATERIALS, INCLUDING, WITHOUT LIMITATION, ANY IMPLIED WARRANTY OF MERCHANTABILITY, ACCURACY, SATISFACTORY QUALITY, FITNESS FOR A PARTICULAR PURPOSE, USABILITY, INTEGRATION OR NON-INFRINGEMENT AND ALL SUCH WARRANTIES ARE HEREBY EXCLUDED BY WILEY AND ITS LICENSORS AND WAIVED BY YOU.

- WILEY shall have the right to terminate this Agreement immediately upon breach of this Agreement by you.

- You shall indemnify, defend and hold harmless WILEY, its Licensors and their respective directors, officers, agents and employees, from and against any actual or threatened claims, demands, causes of action or proceedings arising from any breach of this Agreement by you.

- IN NO EVENT SHALL WILEY OR ITS LICENSORS BE LIABLE TO YOU OR ANY OTHER PARTY OR ANY OTHER PERSON OR ENTITY FOR ANY SPECIAL, CONSEQUENTIAL, INCIDENTAL, INDIRECT, EXEMPLARY OR PUNITIVE DAMAGES, HOWEVER CAUSED, ARISING OUT OF OR IN CONNECTION WITH THE DOWNLOADING, PROVISIONING, VIEWING OR USE OF THE MATERIALS REGARDLESS OF THE FORM OF ACTION, WHETHER FOR BREACH OF CONTRACT, BREACH OF WARRANTY, TORT, NEGLIGENCE, INFRINGEMENT OR OTHERWISE (INCLUDING, WITHOUT LIMITATION, DAMAGES BASED ON LOSS OF PROFITS, DATA, FILES, USE, BUSINESS OPPORTUNITY OR CLAIMS OF THIRD PARTIES), AND WHETHER OR NOT THE PARTY HAS BEEN ADVISED OF THE POSSIBILITY OF SUCH DAMAGES. THIS LIMITATION SHALL APPLY NOTWITHSTANDING ANY FAILURE OF ESSENTIAL PURPOSE OF ANY LIMITED REMEDY PROVIDED HEREIN.

- Should any provision of this Agreement be held by a court of competent jurisdiction to be illegal, invalid, or unenforceable, that provision shall be deemed amended to achieve as nearly as possible the same economic effect as the original provision, and the legality, validity and enforceability of the remaining provisions of this Agreement shall not be affected or impaired thereby.

- The failure of either party to enforce any term or condition of this Agreement shall not constitute a waiver of either party's right to enforce each and every term and condition of this Agreement. No breach under this agreement shall be deemed waived or excused by either party unless such waiver or consent is in writing signed by the party granting such waiver or consent. The waiver by or consent of a party to a breach of any provision of this Agreement shall not operate or be construed as a waiver of or consent to any other or subsequent breach by such other party.

- This Agreement may not be assigned (including by operation of law or otherwise) by you without WILEY's prior written consent.

- Any fee required for this permission shall be non-refundable after thirty (30) days from receipt by the CCC.

- These terms and conditions together with CCC's Billing and Payment terms and conditions (which are incorporated herein) form the entire agreement between you and WILEY concerning this licensing transaction and (in the absence of fraud) supersedes all prior agreements and representations of the parties, oral or written. This Agreement may not be amended except in writing signed by both parties. This Agreement shall be binding upon and inure to the benefit of the parties' successors, legal representatives, and authorized assigns.

- In the event of any conflict between your obligations established by these terms and conditions and those established by CCC's Billing and Payment terms and conditions, these terms and conditions shall prevail.

- WILEY expressly reserves all rights not specifically granted in the combination of (i) the license details provided by you and accepted in the course of this licensing transaction, (ii) these terms and conditions and (iii) CCC's Billing and Payment terms and conditions.

- This Agreement will be void if the Type of Use, Format, Circulation, or Requestor Type was misrepresented during the licensing process.

- This Agreement shall be governed by and construed in accordance with the laws of the State of New York, USA, without regards to such state's conflict of law rules. Any legal action, suit or proceeding arising out of or relating to these Terms and Conditions or the breach thereof shall be instituted in a court of competent jurisdiction in New York County in the State of New York in the United States of America and each party hereby consents and submits to the personal jurisdiction of such court, waives any objection to venue in such court and consents to service of process by registered or certified mail, return receipt requested, at the last known address of such party.

WILEY OPEN ACCESS TERMS AND CONDITIONS

Wiley Publishes Open Access Articles in fully Open Access Journals and in Subscription journals offering Online Open. Although most of the fully Open Access journals publish open access articles under the terms of the Creative Commons Attribution (CC BY) License only, the subscription journals and a few of the Open Access Journals offer a choice of Creative Commons Licenses. The license type is clearly identified on the article.

The Creative Commons Attribution License

The Creative Commons Attribution License (CC-BY) allows users to copy, distribute and transmit an article, adapt the article and make commercial use of the article. The CC-BY license permits commercial and non-

Creative Commons Attribution Non-Commercial License
The Creative Commons Attribution Non-Commercial (CC-BY-NC)License permits use, distribution and reproduction in any medium, provided the original work is properly cited and is not used for commercial purposes.(see below)

Creative Commons Attribution-Non-Commercial-NoDerivs License
The Creative Commons Attribution Non-Commercial-NoDerivs License (CC-BY-NC-ND) permits use, distribution and reproduction in any medium, provided the original work is properly cited, is not used for commercial purposes and no modifications or adaptations are made. (see below)
Use by commercial "for-profit" organizations
Use of Wiley Open Access articles for commercial, promotional, or marketing purposes requires further explicit permission from Wiley and will be subject to a fee.
Further details can be found on Wiley Online Library
http://olabout.wiley.com/WileyCDA/Section/id-410895.html

Other Terms and Conditions:

v1.10 Last updated September 2015

Questions? customercare@copyright.com or +1-855-239-3415 (toll free in the US) or +1-978-646-2777.
