# Supplementary material for: 50 years of scanning electron microscopy of bone—a comprehensive overview of the important discoveries made and insights gained into bone material properties in health, disease, and taphonomy
Source: Bone Res. 2019 May 22;7:15. doi: 10.1038/s41413-019-0053-z (PMC6531483; doi:10.1038/s41413-019-0053-z)
Supplement: Supplementary file 12 — Permission to reuse content [file 41413_2019_53_MOESM12_ESM.pdf]

SPRINGER NATURE LICENSE
TERMS AND CONDITIONS

Mar 02, 2019

This Agreement between Furqan A Shah ("You") and Springer Nature ("Springer Nature") consists of your license details and the terms and conditions provided by Springer Nature and Copyright Clearance Center.

License Number	4541051011826
License date	Mar 02, 2019
Licensed Content Publisher	Springer Nature
Licensed Content Publication	Calcified Tissue International
Licensed Content Title	Microstructural Classification of Resorption Lacunae and Perforations in Human Proximal Femora
Licensed Content Author	C. Gentzsch, G. Delling, E. Kaiser
Licensed Content Date	Jan 1, 2003
Licensed Content Volume	72
Licensed Content Issue	6
Type of Use	Journal/Magazine
Requestor type	academic/university or research institute
Format	print and electronic
Portion	figures/tables/illustrations
Number of figures/tables/illustrations	1
Will you be translating?	no
Circulation/distribution	5,001 to 10,000
Author of this Springer Nature content	no
Title of new article	50 years of scanning electron microscopy of bone – A comprehensive overview of the important discoveries made and insights gained into bone material properties in health, disease, and taphonomy
Lead author	Furqan A. Shah, Krisztina Ruscsák, Anders Palmquist
Title of targeted journal	Bone Research
Publisher	Springer Nature
Expected publication date	Aug 2019
Portions	Figure 2
Requestor Location	Furqan A Shah<br>Department of Biomaterials<br>Arvid Wallgrens Backe 20<br>Box 412<br>Göteborg, 405 30<br>Sweden<br>Attn: Furqan A Shah
Billing Type	Invoice
Billing Address	Furqan A Shah

Department of Biomaterials
Arvid Wallgrens Backe 20
Box 412
Göteborg, Sweden 405 30
Attn: Furqan A Shah

Total 0.00 USD

Terms and Conditions

Springer Nature Terms and Conditions for RightsLink Permissions

Springer Nature Customer Service Centre GmbH (the Licensor) hereby grants you a non-exclusive, world-wide licence to reproduce the material and for the purpose and requirements specified in the attached copy of your order form, and for no other use, subject to the conditions below:

1. The Licensor warrants that it has, to the best of its knowledge, the rights to license reuse of this material. However, you should ensure that the material you are requesting is original to the Licensor and does not carry the copyright of another entity (as credited in the published version).

   If the credit line on any part of the material you have requested indicates that it was reprinted or adapted with permission from another source, then you should also seek permission from that source to reuse the material.

2. Where **print only** permission has been granted for a fee, separate permission must be obtained for any additional electronic re-use.

3. Permission granted **free of charge** for material in print is also usually granted for any electronic version of that work, provided that the material is incidental to your work as a whole and that the electronic version is essentially equivalent to, or substitutes for, the print version.

4. A licence for 'post on a website' is valid for 12 months from the licence date. This licence does not cover use of full text articles on websites.

5. Where **'reuse in a dissertation/thesis'** has been selected the following terms apply: Print rights of the final author's accepted manuscript (for clarity, NOT the published version) for up to 100 copies, electronic rights for use only on a personal website or institutional repository as defined by the Sherpa guideline (www.sherpa.ac.uk/romeo/).

6. Permission granted for books and journals is granted for the lifetime of the first edition and does not apply to second and subsequent editions (except where the first edition permission was granted free of charge or for signatories to the STM Permissions Guidelines http://www.stm-assoc.org/copyright-legal-affairs/permissions/permissions-guidelines/), and does not apply for editions in other languages unless additional translation rights have been granted separately in the licence.

7. Rights for additional components such as custom editions and derivatives require additional permission and may be subject to an additional fee. Please apply to Journalpermissions@springernature.com/bookpermissions@springernature.com for these rights.

8. The Licensor's permission must be acknowledged next to the licensed material in print. In electronic form, this acknowledgement must be visible at the same time as the figures/tables/illustrations or abstract, and must be hyperlinked to the journal/book's homepage. Our required acknowledgement format is in the Appendix below.

9. Use of the material for incidental promotional use, minor editing privileges (this does not include cropping, adapting, omitting material or any other changes that affect the meaning, intention or moral rights of the author) and copies for the disabled are permitted under this licence.

10. Minor adaptations of single figures (changes of format, colour and style) do not require the Licensor's approval. However, the adaptation should be credited as shown in Appendix

below.

Appendix — Acknowledgements:

For Journal Content:
Reprinted by permission from [**the Licensor**]: [**Journal Publisher** (e.g. Nature/Springer/Palgrave)] [**JOURNAL NAME**] [**REFERENCE CITATION** (Article name, Author(s) Name), [**COPYRIGHT**] (year of publication)

For **Advance Online Publication papers:**
Reprinted by permission from [**the Licensor**]: [**Journal Publisher** (e.g. Nature/Springer/Palgrave)] [**JOURNAL NAME**] [**REFERENCE CITATION** (Article name, Author(s) Name), [**COPYRIGHT**] (year of publication), advance online publication, day month year (doi: 10.1038/sj.[JOURNAL ACRONYM].)

For Adaptations/Translations:
Adapted/Translated by permission from [**the Licensor**]: [**Journal Publisher** (e.g. Nature/Springer/Palgrave)] [**JOURNAL NAME**] [**REFERENCE CITATION** (Article name, Author(s) Name), [**COPYRIGHT**] (year of publication)

Note: For any republication from the British Journal of Cancer, the following credit line style applies:

Reprinted/adapted/translated by permission from [**the Licensor**]: on behalf of Cancer Research UK: : [**Journal Publisher** (e.g. Nature/Springer/Palgrave)] [**JOURNAL NAME**] [**REFERENCE CITATION** (Article name, Author(s) Name), **[COPYRIGHT]** (year of publication)

For **Advance Online Publication** papers:
Reprinted by permission from The [**the Licensor**]: on behalf of Cancer Research UK: [**Journal Publisher** (e.g. Nature/Springer/Palgrave)] [**JOURNAL NAME**] [**REFERENCE CITATION** (Article name, Author(s) Name), [**COPYRIGHT**] (year of publication), advance online publication, day month year (doi: 10.1038/sj. [JOURNAL ACRONYM])

For Book content:
Reprinted/adapted by permission from [**the Licensor**]: [**Book Publisher** (e.g. Palgrave Macmillan, Springer etc) [**Book Title**] by [**Book author**(s)] [**COPYRIGHT**] (year of publication)

Other Conditions:

Version 1.1

Questions? customercare@copyright.com or +1-855-239-3415 (toll free in the US) or +1-978-646-2777.
