# Supplementary material for: 50 years of scanning electron microscopy of bone—a comprehensive overview of the important discoveries made and insights gained into bone material properties in health, disease, and taphonomy
Source: Bone Res. 2019 May 22;7:15. doi: 10.1038/s41413-019-0053-z (PMC6531483; doi:10.1038/s41413-019-0053-z)
Supplement: Supplementary file 14 — Permission to reuse content [file 41413_2019_53_MOESM14_ESM.pdf]

Copyright Clearance Center RightsLink

Home | Account Info | Help

ACS Publications
Most Trusted. Most Cited. Most Read.

Title: High-Resolution Visualization of the Osteocyte Lacuno-Canalicular Network Juxtaposed to the Surface of Nanotextured Titanium Implants in Human

Author: Furqan A. Shah, Xiaoyue Wang, Peter Thomsen, et al

Publication: ACS Biomaterials Science & Engineering

Publisher: American Chemical Society

Date: May 1, 2015

Copyright © 2015, American Chemical Society

Logged in as:
Furqan Shah
Account #:
3000820029
LOGOUT

PERMISSION/LICENSE IS GRANTED FOR YOUR ORDER AT NO CHARGE

This type of permission/license, instead of the standard Terms & Conditions, is sent to you because no fee is being charged for your order. Please note the following:

- Permission is granted for your request in both print and electronic formats, and translations.
- If figures and/or tables were requested, they may be adapted or used in part.
- Please print this page for your records and send a copy of it to your publisher/graduate school.
- Appropriate credit for the requested material should be given as follows: "Reprinted (adapted) with permission from (COMPLETE REFERENCE CITATION). Copyright (YEAR) American Chemical Society." Insert appropriate information in place of the capitalized words.
- One-time permission is granted only for the use specified in your request. No additional uses are granted (such as derivative works or other editions). For any other uses, please submit a new request.

If credit is given to another source for the material you requested, permission must be obtained from that source.

BACK | CLOSE WINDOW

Copyright © 2019 Copyright Clearance Center, Inc. All Rights Reserved. Privacy statement. Terms and Conditions.
Comments? We would like to hear from you. E-mail us at customercare@copyright.com
